# The Nrf2 antioxidant defense system in intervertebral disc degeneration: Molecular insights

**DOI:** 10.1038/s12276-022-00829-6

**Published:** 2022-08-17

**Authors:** Qian Xiang, Yongzhao Zhao, Jialiang Lin, Shuai Jiang, Weishi Li

**Affiliations:** 1 Department of Orthopaedics, Peking University Third Hospital, Beijing, China; 2 Engineering Research Center of Bone and Joint Precision Medicine, Ministry of Education, Beijing, China; 3 Beijing Key Laboratory of Spinal Disease Research, Beijing, China

**Keywords:** Molecular biology, Cell biology

## Abstract

Intervertebral disc degeneration (IDD) is a common degenerative musculoskeletal disorder and is recognized as a major contributor to discogenic lower back pain. However, the molecular mechanisms underlying IDD remain unclear, and therapeutic strategies for IDD are currently limited. Oxidative stress plays pivotal roles in the pathogenesis and progression of many age-related diseases in humans, including IDD. Nuclear factor E2-related factor 2 (Nrf2) is a master antioxidant transcription factor that protects cells against oxidative stress damage. Nrf2 is negatively modulated by Kelch-like ECH-associated protein 1 (Keap1) and exerts important effects on IDD progression. Accumulating evidence has revealed that Nrf2 can facilitate the transcription of downstream antioxidant genes in disc cells by binding to antioxidant response elements (AREs) in promoter regions, including heme oxygenase-1 (HO-1), glutathione (GSH), superoxide dismutase (SOD), catalase (CAT), and NADPH quinone dehydrogenase 1 (NQO1). The Nrf2 antioxidant defense system regulates cell apoptosis, senescence, extracellular matrix (ECM) metabolism, the inflammatory response of the nucleus pulposus (NP), and calcification of the cartilaginous endplates (EP) in IDD. In this review, we aim to discuss the current knowledge on the roles of Nrf2 in IDD systematically.

## Intervertebral disc degeneration: Boosting regulatory protein may enhance treatment

Insights into the activity of a protein that regulates gene expression and protects cells against oxidative stress could yield novel treatments for lower back pain. Intervertebral disc degeneration (IDD) is a common cause of lower back pain, but the molecular mechanisms underlying IDD are unclear, meaning treatment options are limited. Oxidative stress is implicated in IDD, and scientists have begun exploring the role of nuclear factor E2-related factor 2 (Nrf2), a master regulator of the body’s antioxidant responses, in regulating IDD progression. In a review of recent research, Weishi Li at Peking University Third Hospital, Beijing, China, and co-workers point out that boosting the activity of Nrf2-related signaling pathways alleviates oxidative stress in intervertebral disc cells. The researchers suggest that therapies based on non-coding RNAs may prove valuable in activating Nrf2 in IDD patients.

## Introduction

Lower back pain (LBP) has become a profoundly debilitating and increasingly prevalent disorder, causing a heavy socioeconomic burden worldwide^1^. The leading cause of LBP is intervertebral disc degeneration (IDD)^2^. However, the pathogenesis of IDD is associated with multiple complex factors, including genetic, epigenetic, and environmental factors, and the knowledge about the molecular mechanisms underlying IDD remains elusive^3,4^. The clinical treatments for IDD are limited to surgery, pharmacological or other nonpharmacological interventions to relieve the symptoms, and more effective therapeutic strategies to address the underlying pathology are needed for this degenerative spine disorder^5^. Therefore, a better understanding of the molecular signaling involved in IDD has been a research focus, which may help to develop novel therapeutic targets for the successful treatment of IDD^6–9^.

Redox homeostasis is crucial for the physiological maintenance of many cellular processes, and dysregulation of redox homeostasis is closely associated with various pathological conditions affecting human health^10^. Oxidative stress is described as the disruption of redox homeostasis, which occurs when the balance between reactive oxygen species (ROS) production and the scavenging activity of the antioxidant system becomes dysregulated^11^. Excessive accumulation of ROS induces oxidative stress, which can cause damage to biological macromolecules such as carbohydrates, lipids, nucleic acids, and proteins, impairing the regular functional integrity of cells in the body^12^. Accumulating evidence has revealed the roles played by oxidative stress in the pathogenesis of various human diseases, especially age-related disorders such as degenerative musculoskeletal diseases^13–15^. Degenerated disks exhibit oxidative stress as well as increased oxidation product levels, contributing to the development of IDD^16^. Importantly, mounting evidence has revealed that therapies targeting oxidative stress might effectively alleviate or prevent IDD progression^17^.

Nuclear factor E2-related factor 2 (Nrf2) is a master endogenous antioxidant transcription factor that has been increasingly reported to play crucial roles in protecting cells against oxidative stress^18^. Physiologically, Nrf2 is critical for the expression of antioxidative genes, cytoprotective enzymes, and export transporters, which constitute an antioxidant defense system that maintains intracellular redox homeostasis^19^. The activation of Nrf2 signaling is negatively regulated by Kelch-like ECH-associated protein 1 (Keap1), which functions as a redox sensor for ROS and electrophiles^20,21^. Under resting conditions, the activity of Nrf2 is tightly controlled by Keap1, which mediates ubiquitination-dependent proteasomal degradation of Nrf2 in the cytoplasm. In the presence of oxidative stress, Keap1 undergoes a conformational change and releases Nrf2, which moves to the nucleus, resulting in the initiation of the transcription of multiple antioxidant genes^22,23^. Nrf2 signaling is considered a central hub that modulates redox homeostasis in cells, and aberrant Keap1-Nrf2 signaling is functionally involved in the pathology of many diseases^24–26^. Interestingly, increasing evidence has revealed the crucial roles played by the Nrf2 signaling pathway in protecting against IDD progression^27–29^. To our knowledge, no systematic review has yet summarized the involvement of Nrf2 in disc degeneration diseases. Therefore, in this review, we synthesize and evaluate the results from the available literature and comprehensively discuss the roles of the Nrf2 antioxidant defense system in IDD.

## Pathophysiology of IDD and oxidative stress

Situated between the vertebral bones, each intervertebral disc (IVD) is made of fibro-cartilaginous tissues and is one of the most important structures of the spine. The IVD can distribute the axial compressive load transmitted from the vertebral bodies and enables physiological lateral and rotational flexibility of the spine^30^. Anatomically, the disc consists of three major parts: the hydrated gel-like nucleus pulposus (NP) in the center, elastic annulus fibrosus (AF) surrounding the NP, and cartilaginous endplates (EP) on the inferior and superior sides^31^. Oxidative stress, compressive overload, nutrient stress, enhanced inflammation, and other factors can act on these parts and stimulate aberrant cellular responses and progressive structural deficiency, leading to disc degeneration^32^. IDD is characterized by a loss of centrally situated NP cells, which are replaced with cells with a fibroblast-like phenotype^33^. Another typical pathological change in disc degeneration is accelerated extracellular matrix (ECM) degradation, such as decreased deposition of type II collagen (Col II) and aggrecan, which is caused by imbalanced anabolism and catabolism^34^. Additionally, cellular senescence and programmed cell death induced by inflammatory responses or other factors in the disc significantly contribute to the pathological changes during the complicated process of IDD^9^.

**Fig. 1:**
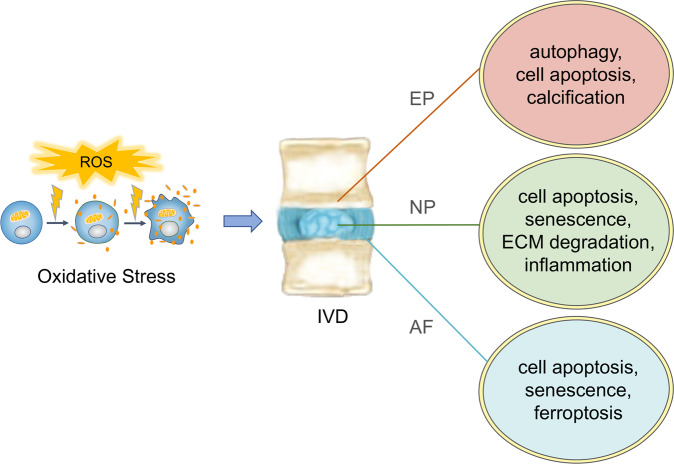
The effects of oxidative stress on disc cells during IDD pathogenesis. Excessive ROS accumulation exerts important effects on the three major types of IVD cells. Oxidative stress induces autophagy, apoptosis, and calcification of EP chondrocytes, while autophagy can act as a protective response to oxidative damage. Oxidative stress promotes cell apoptosis, senescence, ECM degradation, and inflammation response of disc NP cells. Oxidative stress induces cellular senescence, apoptosis, and ferroptosis in disc AF cells.

Oxidative stress is a critical mediator in the initiation and progression of IDD. Oxidative stress occurs when the balance between ROS production and the scavenging activity of the antioxidant defense system is disrupted^11^. Excessive ROS accumulation can induce oxidative stress, which causes damage to the integrity and regular function of cells^35^. Accumulating evidence has suggested that oxidative stress exerts significant effects on cell fate and function and is closely related to cell viability, senescence, programmed cell death, matrix metabolism, and signaling network transduction of disc cells within the IVD^27,36–38^. Previous studies have reported that aged and degenerated disks exhibit decreased antioxidant activity and elevated concentrations of oxidation products during IDD development^16,17^. Excessive ROS accumulation and dysfunction of the antioxidant defense system induce cell apoptosis and senescence and trigger inflammatory responses in disc NP cells, accelerating IDD progression^39^ (Fig. 1). Redox homeostasis in the disc also plays a crucial role in the ECM anabolism and catabolism balance, and oxidative stress has been found to promote ECM degradation by interacting with various important signaling pathways in NP cells, including NF-κB signaling, p38/MAPK signaling, and the Nrf2/ARE signaling pathway^17,40,41^. Moreover, the annulus fibrosus is a crucial part of the IVD, and oxidative stress is involved in the cell senescence, apoptosis, and ferroptosis of disc AF cells in the pathogenesis of IDD^37,42–44^. Disc EP degeneration is another critical contributor to IDD initiation because it hinders the nutrient supply to the NP and leads to disrupted disc homeostasis. It has been demonstrated that oxidative stress can induce autophagy, apoptosis, and calcification of endplate chondrocytes to modulate the EP degeneration process^38,45–47^. Therefore, elucidating the key molecular mechanisms of oxidative stress in the disc might lead to effective therapeutic strategies for IDD.

## Nrf2 mediated antioxidant defense in IDD

Nuclear factor E2-related factor 2 (Nrf2), also known as nuclear factor erythroid 2-like 2 (NFE2L2), is a master antioxidant transcription factor encoded by the NFE2L2 gene in humans^18^. The Nrf2 protein is composed of approximately 605 amino acid residues and possesses seven highly conserved domains, namely, Neh1 to Neh7. Specifically, the Neh2 domain in Nrf2 participates in binding with the Keap1 homodimer and the degradation of Nrf2^48^. The Neh2 domain contains two conserved motifs, DLG and ETGE, with an intervening sequence possessing seven lysine residues that can be ubiquitinated. DLG and ETGE are both associated with the interaction between Nrf2 and Keap1 homodimer. Physiologically, the activation of Nrf2 is regulated by Keap1, which functions as a cysteine-rich oxidative stress sensor. Keap1 is a substrate adaptor protein for the Cullin3 (Cul3)-containing E3 ubiquitin (Ub) ligase complex and is a cytosolic protein that negatively modulates Nrf2 activity^49^. Structurally, the Keap1 peptide is composed of 624 amino acid residues and possesses five functional regions, namely, the N-terminal region (NTR), intervening region (IVR), Broad complex Tramtrack and Bric-a-Brac (BTB) domain, double glycine repeat (DGR) domain and C-terminal region (CTR). The BTB domain is associated with the formation of the Keap1 homodimer, and the DGR and CTR domains (collectively known as the DC region) are involved in the interaction of Keap1 with Nrf2^50^. The ubiquitin-proteasome system (UPS) is responsible for protein quality control and degradation and plays key roles in the maintenance of intracellular protein homeostasis^51^. Under unstressed conditions, Keap1 can bind to Nrf2 and target Nrf2 for ubiquitination and subsequent degradation by the proteasome. However, when ROS levels in cells are increased, the cysteine residues of Keap1 are covalently modified, and Keap1 undergoes a conformational change, resulting in blocked ubiquitination of Nrf2 and accumulation of newly synthesized Nrf2^52^. Then, Nrf2 is released into the nucleus, where it forms a heterodimer with small musculoaponeurotic fibrosarcoma (Maf) proteins^53^. Subsequently, Nrf2-Maf binds to the antioxidant response element (ARE) in DNA to promote the transcription of multiple downstream antioxidant genes, including heme oxygenase-1 (HO-1), glutathione (GSH), superoxide dismutase (SOD), catalase (CAT), and NADPH quinone dehydrogenase 1 (NQO1)^48^.

**Fig. 2:**
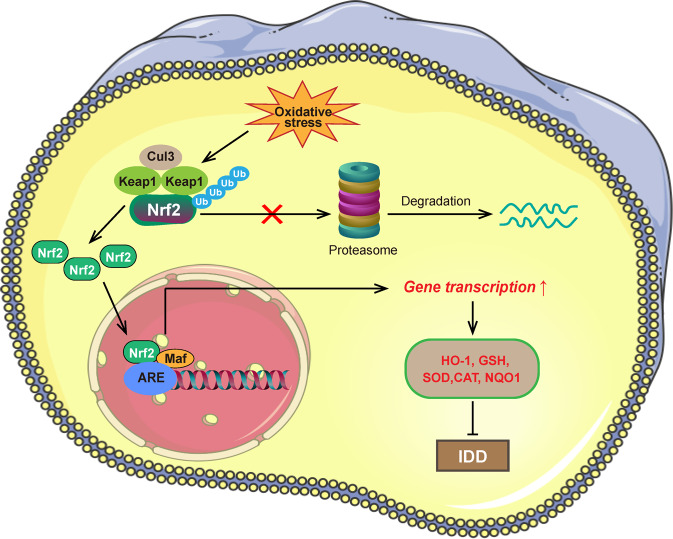
Nrf2 mediates antioxidant defense in IDD. In unstressed conditions, Keap1 binds to the Cul3-containing E3 ubiquitin ligase complex, and two molecules of Keap1 form a homomeric dimer. The Keap1 complex binds to Nrf2 for the ubiquitination and subsequent degradation of Nrf2 by the proteasome. Under conditions of oxidative stress, Keap1 undergoes a conformational change, which leads to blocked ubiquitination of Nrf2 and accumulation of newly synthesized Nrf2. Subsequently, free Nrf2 is translocated to the nucleus, where it forms a heterodimer with small Maf proteins. Then, Nrf2-Maf interacts with the ARE in the promoter regions of DNA to promote the transcription of multiple targeted antioxidant genes, including HO-1, GSH, SOD, CAT, and NQO1. Activating Nrf2 signaling protects against oxidative stress in disc cells to alleviate IDD.

IDD is one of the most common age-related degenerative musculoskeletal disorders. As mentioned above, the pathogenesis of IDD is closely associated with oxidative stress. Nrf2 is a crucial transcription factor that can modulate the cellular oxidative stress response. An increasing number of studies have revealed the important roles played by the Nrf2 antioxidant defense system in preventing IDD progression. Multiple antioxidants, including ulinastatin, dimethyl fumarate, and cyanidin-3-glucoside, have been reported to alleviate oxidative stress in disc NP cells by promoting the activity of the Nrf2-mediated HO-1 signaling pathway^54–57^. Dimethyl fumarate has also been demonstrated to activate Nrf2 to promote the production of GSH in NP cells, which is one of the most important ROS scavengers^55^. Another study reported that Nrf2/HO-1 signaling activated by moracin dramatically promoted the expression levels of SOD and CAT in NP cells induced by LPS challenge^58^. Acacetin and wogonin were also reported to activate the Nrf2 pathway to upregulate the expression of important antioxidant proteins, including HO-1, SOD, and NQO1, to ameliorate IDD progression^59,60^. Interestingly, it has been reported that activating autophagy promoted Nrf2 signaling to upregulate the expression of antioxidant proteins, including SOD1 and SOD2, and thus protect cartilage endplate stem cells against calcification and ECM degradation during IDD^61^. In summary, activating Nrf2 signaling facilitated the transcription of downstream antioxidant genes, including HO-1, GSH, SOD, CAT, and NQO1, to defend against oxidative stress in disc cells. The molecular mechanism of the Nrf2 antioxidant pathway is indicated in Fig. 2.

## Therapeutic potential of targeting Nrf2 signaling in IDD treatment

### Targeting Nrf2 to alleviate apoptosis of NP cells

The highly hydrated NP is the structural and functional center of a disc. Dysregulated NP cell apoptosis causes damage to the normal metabolism in the NP, which disrupts the normal structure and physiological function of the disc and is considered a key contributor to IDD pathogenesis. It has been demonstrated that the apoptosis ratio of NP cells is more than 50% in human degenerative disks, and preventing or alleviating apoptosis of NP cells is a potential effective therapy to treat disc degeneration^32,62^. Accumulating evidence has reported antiapoptotic roles played by Nrf2 activation in various types of human cells^63–65^. Unexpectedly, targeting Nrf2 signaling regulated the apoptosis of NP cells during IDD progression. Long noncoding RNAs (lncRNAs) constitute a common and diverse class of noncoding RNAs (ncRNAs) without protein-coding capacity^66^. Recently, Kang et al.^27^ reported that lncRNA ANPODRT activated Nrf2 signaling to inhibit oxidative stress and apoptosis in human NP cells. Mechanistically, the lncRNA ANPODRT facilitated Nrf2 accumulation and nuclear translocation to activate downstream target genes by disrupting the Keap1-Nrf2 interaction. Moreover, Nrf2 knockdown obliterated the antioxidative and antiapoptotic effects of the lncRNA ANPODRT, indicating that Nrf2 activation is required for the lncRNA ANPODRT to exert protective effects on NP cells. MicroRNAs (miRNAs) comprise another important and large class of short-chain noncoding RNAs that regulate downstream genes by targeting the 3’ untranslated region (3’UTR) posttranscriptionally. A study by Xu et al.^67^ revealed that a miRNA termed miR-141-3p, which was enriched in platelet-rich plasma (PRP)-derived exosomes, activated Keap1-Nrf2 signaling to reverse the cell apoptosis, pyroptosis, and inflammatory response of NP cells stimulated by H_2_O_2_. Mechanistically, miR-141-3p interacted with the 3’UTR of Keap1 mRNA to induce its degradation, thus leading to Nrf2 translocation to the nucleus.

More recently, Hu et al.^28^ reported the critical roles played the Nrf2 agonist tert-butylhydroquinone (TBHQ) in retarding NP cell apoptosis. The results showed that TBHQ rescued TBHP-induced apoptosis and oxidative stress by promoting Nrf2 expression and translocation to the nucleus. Mechanistically, TBHQ resisted oxidative stress by inducing Nrf2 activity and increasing the Sirt3 expression level to maintain mitochondrial homeostasis and enhance mitochondrial autophagy. Furthermore, the authors validated the therapeutic function and mechanism of TBHQ in a rat tail disc degeneration model in vivo. Mitoquinone (MitoQ) is a known mitochondria‐targeted antioxidant that has shown protective effects in various oxidative damage‐related diseases^68^. It has been suggested that MitoQ alleviates sustained mitochondrial dysfunction, oxidative stress, and apoptosis of NP cells by stimulating the Nrf2 antioxidant pathway in vitro and ex vivo^69^. Luo et al.^54^ found that an anti-inflammatory acidic protein extracted from human urine, ulinastatin, ameliorated the apoptosis of human NP cells by activating the Nrf-2/HO-1 signaling pathway and suppressing the NF-κB signaling pathway. Treatment with ulinastatin reversed the expression of the apoptosis-related proteins Bax and cleaved-caspase 3 and the antiapoptosis molecule Bcl-2. Moreover, increasing evidence has revealed other crucial molecular agents that mitigate excessive apoptosis of NP cells by interacting with Nrf2 signaling; these agents include sinapic acid^70^, plumbagin^71^, dimethyl fumarate^55^, luteoloside^72^, CDDO-ethyl amide^73^, cyanidin-3-glucoside^57^, kinsenoside^74^, lycopene^75^, and genistein^76^. Taken together, these studies revealed that activating Nrf2 signaling is a promising strategy to attenuate the apoptosis of NP cells and treat IDD.

### Targeting Nrf2 to inhibit NP cell senescence

Numerous studies have reported that the impairment of NP cell function caused by senescence is a crucial contributor to the dehydration of NP tissue and, more importantly, to the initiation and progression of disc degeneration^7,77,78^. Senescent disc cells are metabolically active and can secrete various inflammatory cytokines, chemokines and matrix proteases, which collectively are known as the senescence-associated secretory phenotype (SASP)^79,80^. These inflammatory factors have been found to disrupt the balance between ECM anabolism and catabolism during IDD. Moreover, the SASP of senescent cells can induce senescence in neighboring nonsenescent cells by paracrine effects, which is referred to as paracrine senescence or secondary senescence^79,81^. The increase in inflammatory factor expression levels as a result of senescence causes a vicious cycle of degeneration and leads to further aggravation of IDD. Obviously, protecting disc NP cells against senescence is conducive to the amelioration of IDD.

In 2019, Cherif et al.^82^ reported that curcumin and o-vanillin exhibited significant senolytic activity in human degenerative disc NP cells. Curcumin, diferuloylmethane, has wide therapeutic benefits via its antioxidative and anti-inflammatory properties^83^, and its main metabolite, o-vanillin (2-hydroxy-3-methoxybenzaldehyde), shows similar effects^84^. This research revealed that curcumin and o-vanillin mediated senolytic effects via Nrf2 signaling and decreased SASP factor secretion by suppressing NF-κB pathway activation. A recent study by Shao et al.^85^ demonstrated that quercetin, a natural senolytic compound, activated Nrf2 signaling to suppress SASP factor expression and the senescence phenotype acquisition by NP cells. Mechanistically, quercetin suppressed IL-1β-induced activation of NF-κB pathway cascades by directly binding to the Keap1-Nrf2 complex. A previous work reported that kinsenoside activated the AKT-ERK1/2-Nrf2 signaling pathway in NP cells to attenuate IDD both in vitro and in vivo^74^. Kinsenoside is an active monomer extracted from *Anoectochilus roxburghii*, a traditional Chinese medicinal herb that exhibits diverse pharmacological actions. Importantly, kinsenoside has been shown to protect NP cells from apoptosis, senescence, and mitochondrial dysfunction in a Nrf2-dependent manner. Polydatin is a resveratrol glucoside that exerts extensive pharmacological antioxidative, anti‐inflammatory, and anti‐aging properties^86^. It has been reported that polydatin rescued mitochondrial dysfunction, suppressed senescence, and preserved ECM homeostasis in nucleus pulposus cells to attenuate IDD progression by promoting Nrf2 activity^87^. In summary, triggering Nrf2 activation to inhibit NP cell senescence is a potential therapeutic strategy for IDD.

### Targeting Nrf2 to regulate ECM metabolism in NP cells

Physiologically, the ECM endows the IVD with elastic and weight-bearing properties, allowing it to absorb compression loads while maintaining flexibility in the spine^88^. The ECM is mainly composed of proteoglycans (mainly aggrecan) and Col II in disc NP tissues^89^. ECM metabolism is generally modulated by degradative enzymes, including matrix metalloproteinases (MMPs) and aggrecanases, and their inhibitors, tissue inhibitors of metalloproteinases (TIMPs)^90,91^. Degenerative disks are biochemically characterized by an imbalanced ECM metabolism of NP cells, implicating attenuated anabolic activities and enhanced catabolic activities in the disc. In this process, excessive degradation of aggrecan and Col II leads to NP dehydration and resorption and a decline in the ability of the cells to resist mechanical loading, thus contributing to IDD progression^92,93^.

A recent study reported that lncRNA NEAT1 overexpression accelerated the ECM degradation of NP cells, while the Nrf2 activator TBHQ partially reversed the effects of the lncRNA NEAT1 on ECM metabolism^41^. These results suggested that the lncRNA NEAT1 ameliorated ECM degradation of NP cells by regulating Nrf2 signaling pathway activation. Dimethyl fumarate is a known agonist of Nrf2-responsive genes and has been applied in certain clinically degenerative diseases^94^. It has been revealed that dimethyl fumarate helped maintain the ECM metabolic balance of human NP cells, mainly by regulating the Nrf2/HO-1 signaling pathway^55^. As mentioned above, the anti-inflammatory acidic protein extracted from human urine, ulinastatin, also protected human NP cells from ECM degradation by activating the Nrf-2/HO-1 signaling pathway and suppressing the NF-κB signaling pathway^54^. Treatment with either curcumin or o-vanillin increased the proteoglycan and type II collagen content and inhibited MMP3 and MMP13 expression in human disc cells. Further experiments suggested that curcumin and o-vanillin promoted ECM synthesis in IVD, which was mediated by the Nrf2 and NF-κB pathways^82^. Furthermore, it has been reported that some other important biologically active components also regulated ECM metabolism in NP cells by targeting Nrf2 signaling; these compounds included cardamonin^95^, sinapic acid^70^, luteoloside^72^, cyanidin-3-glucoside^57^, moracin^58^, acacetin^59^, wogonin^60^, lycopene^75^, genistein^76^, and polydatin^87^. Altogether, these results revealed that targeting Nrf2 signaling to alleviate ECM degradation of NP cells is a potential therapeutic strategy for IDD.

### Targeting Nrf2 to regulate the inflammatory response in NP cells

In the initiation and progression of IDD, inflammation is widely acknowledged as a major characteristic feature^96,97^. Accumulating evidence has demonstrated that excessive inflammatory responses can significantly affect the normal function of NP cells and thus contribute to IDD development^4,32^. Dysregulated expression of proinflammatory cytokines such as interleukin (IL)-1, IL-6, IL-17, and tumor necrosis factor (TNF)-α has been observed in degenerated disc NP tissues and has been involved in the inflammatory response during IDD^98^. These proinflammatory cytokines also play critical roles in the pathophysiological processes of IDD, including NP cell apoptosis, senescence, ECM remodeling, neovascularization, and oxidative stress^4,32,99^. Therefore, regulating the inflammatory microenvironment in NP cells is essential for IDD treatment.

Nrf2 is widely involved in the modulation of the inflammatory response in NP cells. As mentioned above, the study by Xu et al.^67^ revealed that exosomal miR-141-3p activated Keap1-Nrf2 signaling to regulate the inflammatory response of NP cells stimulated by H_2_O_2_. Mechanistically, miR-141-3p directly interacted with the 3′UTR of Keap1 mRNA, causing Keap1 degradation, resulting in Nrf2 translocation to the nucleus, and thus inhibiting proinflammatory cytokine (IL-1β, IL-18, TGF-β, and IL-6) production and secretion by NP cells. It has been reported that the anti-inflammatory acidic protein extracted from human urine, ulinastatin, also dramatically suppressed the expression levels of proinflammatory mediators in human NP cells, including IL-6, TNF-α, iNOS, and COX-2, by activating the Nrf-2/HO-1 signaling pathway and suppressing the NF-κB signaling pathway^54^. Interestingly, studies have shown that cardamonin^95^, sinapic acid^70^, and plumbagin^71^ protected NP cells against inflammation by modulating Nrf2/NF-κB axis activation. The known agonist for the Nrf2-responsive gene dimethyl fumarate has been found to ameliorate NP cell inflammation by promoting the activity of the Nrf2/HO-1 signaling pathway in IDD^55,56^. Moreover, evidence has suggested that some other biologically active components, including moracin^58^, acacetin^59^, and wogonin^60^, regulated the inflammatory response in NP cells by regulating the Nrf2 signaling pathway. Collectively, these data revealed that targeting Nrf2 signaling to regulate the inflammatory response in NP cells may be a promising therapeutic strategy for IDD.

### Targeting Nrf2 to alleviate degeneration and calcification of EP

The human IVD has large vascular channels passing through the cartilaginous endplates at birth. With increasing age, however, these vessels recede, leaving the disc with little direct vascular supply^100^. The IVD becomes the largest avascular organ of the body in adulthood. The cartilaginous endplates that attach the disc to the adjacent vertebral bodies provide the major portal for the diffusion of nutrients into the disc inner tissues^100,101^. Therefore, the integrity of the EP structure is of great significance to the maintenance of homeostasis in the IVD. Histology and pathology have revealed that cartilaginous endplate calcification is a major pathological characteristic of disc degeneration^100,102^. The degeneration and calcification of EP hinder the transport of nutrients and metabolite clearance in IVD and thus impair the survival and functions of disc cells, which is considered a crucial initiating mechanism of IDD^103^.

Recently, Kang et al.^45^ revealed the critical roles of oxidative stress and Nrf2 signaling in the cartilaginous endplate homeostasis of IVD. The authors found that H_2_O_2_ stimulated oxidative stress, mitochondrial dysfunction, and cell apoptosis of human endplate chondrocytes, which were enhanced by Nrf2 knockdown. Moreover, upregulation of Nrf2 expression by polydatin treatment significantly protected endplate chondrocytes against these detrimental H_2_O_2_-induced effects. The study also applied the puncture-induced rat IDD model to validate the beneficial effects of Nrf2 activation on EP and disc degeneration. Interestingly, another study reported that rapamycin, a lipophilic macrolide antibiotic isolated from the actinomycete *Streptomyces hygroscopicus*, activated autophagy-Nrf2 signaling to protect cartilage endplate stem cells against calcification and ECM degradation^61^. Tumor necrosis factor-α (TNF-α) treatment induced oxidative stress, cell senescence and the osteogenic differentiation of cartilage endplate stem cells. Mechanistically, rapamycin-induced autophagy to upregulate antioxidant protein expression, scavenge ROS, alleviate cell senescence and promote the chondrogenic differentiation potential of cartilage endplate stem cells. Moreover, the function of rapamycin-activated autophagy in inhibiting TNF-α-induced EP degeneration was realized through the regulated expression and nuclear translocation of Nrf2. Hence, targeting Nrf2 signaling to alleviate degeneration and calcification of EP might be an effective therapeutic means of IDD intervention. A list of the functional mechanisms of Nrf2 activation and related signaling pathways in IDD treatment is presented in Table 1.

**Table 1:** Functional mechanisms of Nrf2 activation and related signaling pathways in IDD treatment.

Experimental models	Molecular agents	Signaling pathways	Functional mechanisms	References
Human NP cells (in vitro)	LncRNA ANPODRT	Keap1/Nrf2	Inhibit oxidative stress and cell apoptosis	Ref. ^27^
Mouse NP cells (in vitro)	Exosomal miR-141-3p	Keap1/Nrf2	Promote cell proliferation and viability, inhibit cell apoptosis, pyroptosis, and inflammation	Ref. ^67^
Human NP cells (in vitro), rat caudal disc (ex vivo)	Mitoquinone	Keap1/Nrf2	Inhibit oxidative stress, mitochondrial impairment, and cell apoptosis	Ref. ^69^
Human NP cells (in vitro)	LncRNA NEAT1, tert-butylhydroquinone (TBHQ)	Nrf2/ARE	Inhibit ECM degradation	Ref. ^41^
Human NP cells (in vitro), rat tail disc (in vivo)	Quercetin	Nrf2/NF-κB	Inhibit cell senescence	Ref. ^85^
Human NP cells (in vitro)	Ulinastatin	Nrf-2/HO-1/NF-κB	Inhibit oxidative stress, inflammation, apoptosis, and ECM degradation	Ref. ^54^
Rat NP cells (in vitro), rat tail disc (in vivo)	Cardamonin	Nrf2/NF-κB	Inhibit inflammation and ECM degradation	Ref. ^95^
Rat NP cells (in vitro), rat tail disc (in vivo)	Sinapic acid	Nrf2/NF-κB	Inhibit apoptosis, inflammation, and ECM degradation	Ref. ^70^
Rat NP cells (in vitro)	Plumbagin	Nrf2/NF-κB	Inhibit oxidative stress, inflammation, and apoptosis	Ref. ^71^
Rat NP cells (in vitro), rat tail disc (in vivo)	TBHQ	Nrf2/Sirt3	Inhibit oxidative stress and cell apoptosis, promote mitophagy	Ref. ^28^
Human NP cells (in vitro)	Dimethyl fumarate	Nrf2/HO-1	Inhibit oxidative stress, inflammation, ER stress-associated apoptosis, and ECM degradation	Ref. ^55^
Human NP cells (in vitro), mouse tail disc (in vivo)	Dimethyl fumarate	Nrf2/HO-1	Inhibit oxidative stress and inflammation	Ref. ^56^
Rat NP cells (in vitro), rat tail disc (in vivo)	Luteoloside	Nrf2/HO-1	Inhibit apoptosis and ECM degradation	Ref. ^72^
Rabbit NP cells (in vitro)	CDDO-ethyl amide	Nrf2/HO-1	Inhibit oxidative stress and cell apoptosis	Ref. ^73^
Human NP cells (in vitro)	Cyanidin-3-glucoside	Nrf2/HO-1	Inhibit oxidative stress, apoptosis, and ECM degradation	Ref. ^57^
Rat NP cells (in vitro)	Moracin	Nrf-2/HO-1/NF-κB/TGF-β	Inhibit oxidative stress, inflammation, and ECM degradation	Ref. ^58^
Rat NP cells (in vitro), rat tail disc (in vivo)	Kinsenoside	AKT-ERK1/2-Nrf2	Inhibit apoptosis, senescence, and mitochondrial impairment	Ref. ^74^
Mouse NP cells (in vitro), mouse tail disc (in vivo)	Keap1 siRNA	Keap1/Nrf2/p62	Promote autophagy, inhibit oxidative stress	Ref. ^104^
Rat NP cells (in vitro), rat tail disc (in vivo)	Wogonin	Nrf2/ARE	Inhibit inflammation and ECM degradation	Ref. ^60^
Rat NP cells (in vitro), rat tail disc (in vivo)	Acacetin	Nrf2	Inhibit inflammation and ECM degradation	Ref. ^59^
Human NP cells (in vitro)	Sulforaphane	Nrf2	Inhibit cell apoptosis and oxidative stress	Ref. ^105^
Human NP cells (in vitro)	Lycopene	Nrf2	Inhibit cell apoptosis and ECM degradation	Ref. ^75^
Human NP cells (in vitro)	Curcumin and o-Vanillin	Nrf2	Inhibit cell senescence and ECM degradation	Ref. ^82^
Rat NP cells (in vitro), rat tail disc (in vivo)	Genistein	Nrf2	Inhibit cell apoptosis and ECM degradation	Ref. ^76^
Rat NP cells (in vitro), rat tail disc (in vivo)	Polydatin	Nrf2	Inhibit mitochondrial impairment, cell senescence, and ECM degradation	Ref. ^87^
Human endplate chondrocytes (in vitro), rat tail disc (in vivo)	Polydatin	Nrf2	Inhibit oxidative stress, mitochondrial impairment, and cell apoptosis	Ref. ^45^
Mouse cartilage endplate stem cells (in vitro), mouse tail disc (in vivo)	Rapamycin	Keap1/Nrf2	Inhibit calcification and ECM degradation	Ref. ^61^

## Conclusions and perspectives

**Fig. 3:**
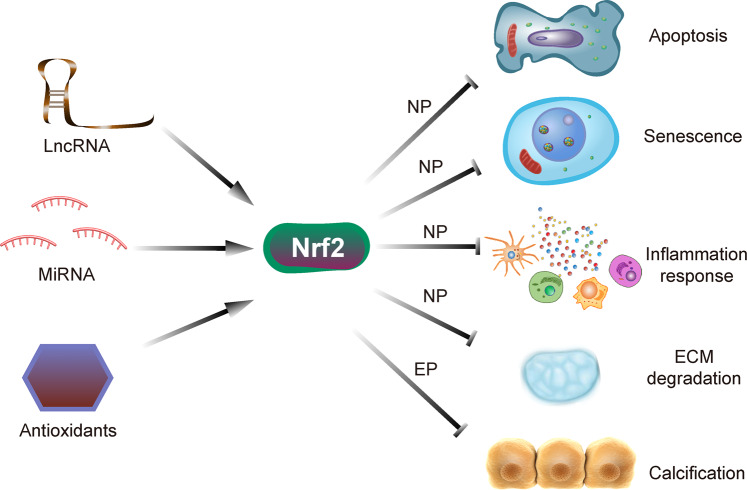
Schematic diagram showing the major mechanisms by which Nrf2 is activated to prevent IDD. Noncoding RNAs, such as lncRNAs and miRNAs, and other antioxidants can activate Nrf2 to alleviate IDD progression by inhibiting cell apoptosis, senescence, inflammation response, and ECM degradation in NP cells and alleviating the degeneration and calcification of EP.

Oxidative stress has been demonstrated to play pivotal roles in the initiation and progression of a plethora of age-related diseases in humans. IDD is one of the most prevalent degenerative musculoskeletal disorders, and its pathogenesis is closely associated with oxidative stress. Nrf2 is a master antioxidant transcription factor and protects cells against oxidative stress damage, similar to its role in disc cells. As mentioned above, certain noncoding RNAs, including lncRNAs and miRNAs, and important antioxidants, such as bioactive compounds and small molecules from natural products, can activate Nrf2 signaling to alleviate IDD progression. Activating Nrf2 helps maintain the structural and functional integrity of IVD by inhibiting cell apoptosis, senescence, inflammation response, and ECM degradation of NP cells and alleviating degeneration and calcification of EP (Fig. 3). Therefore, targeting the Nrf2 antioxidant defense system is an effective therapeutic strategy for IDD. Although pharmacological Nrf2 activators have proven the benefits of defending against oxidative stress to prevent IDD progression in vitro and in vivo models, further investigations are needed to discover the details of the underlying molecular mechanism. In addition, mitochondria are intimately related to oxidative stress, as they are the main sources of intracellular ROS. Whether and how Nrf2 signaling regulates mitochondrial quality control in IDD might be a difficult but interesting area to address in the future. In addition, crosstalk between Nrf2 and important signaling pathways or cellular protective mechanisms, such as autophagy, is evident. Therefore, there remains a need for further systematic studies to clarify the multiple connected and intertwined mechanisms involved in IDD.

## Data Availability

The data used to support this study were included in the article.
